# Lymphocytic interstitial pneumonia and pulmonary amyloidosis in Sjögren's syndrome

**DOI:** 10.1590/0100-3984.2017.0212

**Published:** 2019

**Authors:** Eurípedes Barsanulfo de Paula Avelino, Leonardo Verza, Tércia Neves, Rubens Chojniak, Marcos Duarte Guimarães

**Affiliations:** 1 A.C.Camargo Cancer Center, São Paulo, SP, Brazil

Dear Editor,

A 72-year-old male patient who was a former light smoker presented with a complaint of dyspnea. In 2014, he had been diagnosed with Sjögren’s syndrome during investigation of thrombocytopenia identified on a routine laboratory test. An X-ray performed prior to transurethral resection of the prostate showed pulmonary nodules. Further evaluation with computed tomography (CT) of the chest revealed multiple thin-walled pulmonary cysts in the peribronchovascular and subpleural regions of both lungs, predominantly in the middle and lower lung fields, together with solid, irregular, partially calcified nodules, some in close proximity to the cysts (Figure 1). A biopsy of the largest nodule revealed fragments of lung parenchyma with lymphocytic infiltrate and proteinaceous fibrin filling the alveolar spaces, with degenerated red blood cells (ghost cells), sometimes forming hyaline membranes. Complementary analysis of the material showed an light chain amyloidosis (kappa) peptide profile.

**Figure 1:**
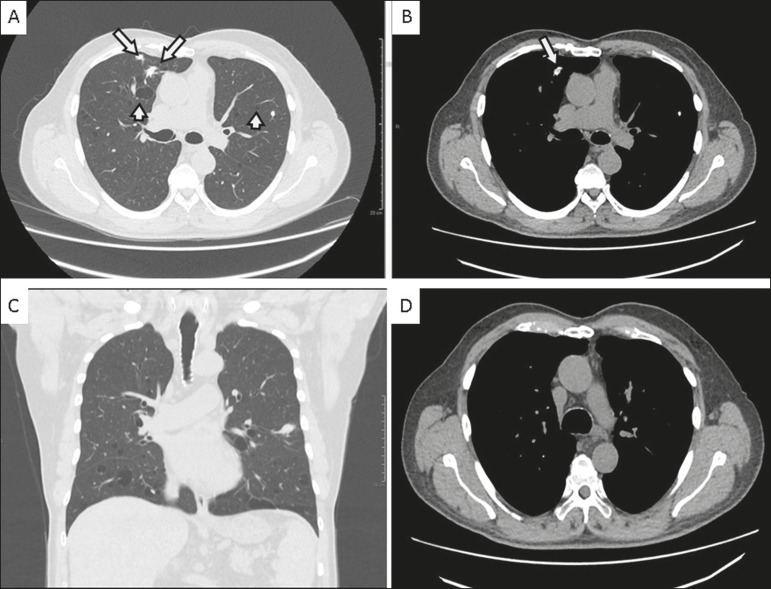
**A,B:** Axial CT images of the chest at the level of the main bronchi, with lung and mediastinal window settings (**A** and **B**, respectively), showing thin-walled cysts in the peribronchovascular and subpleural regions (short arrows), as well as irregular, calcified pulmonary nodules (long arrows). Note also the preservation of the thickness of the bronchial wall. **C:** Coronal CT scan of the chest, with lung window settings, showing sparse nodules and cystic predominance in the middle and lower lung fields. **D:** Axial CT scan of the chest, with mediastinal window settings, highlighting the absence of mediastinal lymphadenopathy and the preservation of the thickness of the tracheal wall.

Sjögren’s syndrome is an autoimmune disease in which lymphocytes attack the glands that generate saliva and tears^(1)^. Many patients with Sjögren’s syndrome develop interstitial lung diseases such as lymphocytic interstitial pneumonia (LIP), amyloidosis, follicular bronchiolitis, and even lymphoma^(1,2)^. On CT, LIP can manifest as ground-glass opacity or consolidations, as well as septal thickening, centrilobular nodules, and cysts^(3)^. Cysts are believed to be formed by air trapping caused by a check-valve mechanism, with airway dilation distal to bronchiolar obstruction caused by lymphocytic infiltrate, and can be the only residual findings in chronic cases^(3,4)^.

Amyloidosis occurs due to excessive formation and deposition of certain proteins in an abnormal fibrillar pattern, resulting in malfunction of the affected organ^(3,4)^. Pulmonary nodular amyloidosis typically manifests as multiple nodules, of varying attenuation, which can cavitate^(3,4)^. Some are associated with mucosa-associated lymphoid tissue lymphoma^(3,4)^. In the clinical context of Sjögren’s syndrome, calcification within a nodule is more consistent with amyloid nodules^(2)^. More rarely, amyloidosis can also lead to the formation of pulmonary cysts, of varying sizes^(3,4)^. The mechanism of cyst formation is uncertain and is believed to involve a check-valve mechanism secondary to narrowing of the airways, caused by the accumulation of inflammatory or amyloid cells or by capillary rupture due to amyloid deposition with alveolar destruction and cyst formation^(3)^. In alveolar-septal amyloidosis, the CT findings include septal thickening and ground-glass opacity, whereas CT shows circumferential thickening of the tracheobronchial wall in the more common form of the disease^(3-5)^.

The prognosis for patients with amyloidosis and LIP varies, the condition resolving in some patients, whereas it progresses to pulmonary fibrosis and respiratory failure in others^(3)^. Although there is no cure, corticosteroids can be used for symptom relief^(3)^. In rare cases, such as the one presented here, Sjögren’s syndrome, amyloidosis, and LIP can coexist. In patients with Sjögren’s syndrome, distinguishing cystic amyloidosis from LIP with amyloidosis is a diagnostic challenge. The diagnosis of LIP with amyloidosis should be ruled out before attributing the cysts exclusively to amyloidosis, because the CT finding of multiple calcified nodules and cysts in patients with Sjögren’s syndrome typically suggests a diagnosis of pulmonary amyloidosis and lymphoproliferative disease, which should be born in mind by the attending physician.

## References

[1] Gupta N, Wikenheiser-Brokamp KA, Fischer A (2016). Diffuse cystic lung disease as the presenting manifestation of Sjögren syndrome. Ann Am Thorac Soc.

[2] Zamora AC, White DB, Sykes AM (2016). Amyloid-associated cystic lung disease. Chest.

[3] Gupta N, Vassallo R, Wikenheiser-Brokamp KA (2015). Diffuse cystic lung disease. Part II. Am J Respir Crit Care Med.

[4] Francisco FAF, Souza Jr AS, Zanetti G (2015). Multiple cystic lung disease. Eur Respir Rev.

[5] Torres PPTS, Rabahi M, Pinto SA (2017). Primary tracheobronchial amyloidosis. Radiol Bras.

